# Intraocular Pressure Fluctuation during Aerobic Exercise at Different Exercise Intensities

**DOI:** 10.3390/healthcare10071196

**Published:** 2022-06-26

**Authors:** Toshihiro Kawae, Takuo Nomura, Daisuke Iwaki, Yuki Nakashima, Kenichi Fudeyasu, Hiroaki Kataoka, Tomoyasu Ishiguro, Hiroaki Kimura

**Affiliations:** 1 Department of Physical Therapy, Makuhari Human Care Faculty, Tohto University, Hibino 1-1, Mihama-ku, Chiba City 261-0021, Chiba, Japan; tomoyasu.ishiguro@tohto.ac.jp; 2 Department of Rehabilitation Sciences, Kansai University of Welfare Sciences, Kashiwara City 582-0026, Osaka, Japan; tnomura@tamateyama.ac.jp; 3 Division of Rehabilitation, Department of Clinical Practice and Support, Hiroshima University Hospital, Kasumi 1-2-3, Minami-ku, Hiroshima City 734-8551, Hiroshima, Japan; dai-iwaki@hiroshima-u.ac.jp (D.I.); ynakashi@hiroshima-u.ac.jp (Y.N.); fudeyasu@hiroshima-u.ac.jp (K.F.); 4 Department of Physical Therapy, Faculty of Health Sciences, Okayama Healthcare Professional University, Daiku 3-2-18, Kitaku, Okayama City 700-0913, Okayama, Japan; h.kataoka59@gmail.com; 5 Department of Rehabilitation, Hiroshima University Hospital, Kasumi 1-2-3, Minami-ku, Hiroshima City 734-8551, Hiroshima, Japan; lunalunalunamusic@gmail.com

**Keywords:** intraocular pressure, aerobic exercise, exercise intensities

## Abstract

Few studies have examined the effects of different aerobic-exercise intensities on intraocular-pressure (IOP) changes. This may be important for eye diseases that are impacted by IOP or its fluctuation, including glaucoma, and diabetes that is complicated by diabetic retinopathy. We investigated the effects of low-, moderate-, and high-intensity exercise on IOP in healthy subjects. A submaximal cardiopulmonary exercise test was performed in 18 healthy male subjects, and the maximal oxygen uptake was calculated. The subjects then exercised for 20 min at 30%, 50%, and 70% ·VO_2_ of maximal oxygen uptake, and their IOP was measured at rest and every 5 min during exercise. Oxygen uptake was monitored using an expiratory gas analyzer during exercise to maintain accurate exercise intensity and adjust exercise load. Oxygen uptake during exercise was significantly higher at all intensities from 5 to 20 min than at rest. IOP was significantly lower at 70% exercise intensity from 5 to 20 min than at rest. A negative correlation existed between IOP and ·VO_2_. IOP remained unchanged during low- and moderate-intensity exercise but significantly declined during high-intensity exercise compared with that at rest. Although various factors, such as β-blockers, are involved in IOP decline at rest, a different mechanism is involved in IOP decline during exercise.

## 1. Introduction

The number of people who have diabetes worldwide was estimated to be 537 million in 2021, corresponding to approximately one in 10 people. The prevalence of diabetes is predicted to increase to 643 million by 2030 and 783 million by 2045 [1]. Therefore, there is an urgent need to prevent the onset and severity of diabetes worldwide. Exercise is a well-established treatment for type 2 diabetes, as it improves glycemic control [2], obesity [3], and the accumulation of visceral fat [4]. Aerobic and resistance exercises are the recommended types of exercise [5]. Complications of diabetes mellitus include neuropathy, nephropathy, and retinopathy. In a recent study of 121 patients with diabetic nephropathy, an intervention that included dietary guidance and brisk walking for at least 300 min per week showed no improvement in HbA1c, a measure of glycemic control, compared with that in control subjects. However, body mass index (BMI), albumin-to-creatinine ratio, serum and urinary 8-OHdG, and plasma TGF-β1 were significantly lower in the intervention group [6]. Robinson et al. investigated 256 patients with chronic kidney disease to determine the association between physical activity and estimated glomerular filtration rate (eGFR) [7]. Their study revealed that each additional 60 min of physical activity per week showed an annual 0.5% decline in eGFR, suggesting that interventions including exercise therapy may delay kidney damage in patients with diabetes. 

In one study, a 10-week intervention including aerobic and resistance exercise in 20 patients with diabetic neuropathy resulted in reduced adiposity and altered resting tissue properties in the lower leg compared with those in the control group. This study also reported that continuous exercise therapy improved muscle quality in patients with diabetic neuropathy [8]. Furthermore, after 16 weeks of aerobic exercise, perceived pain interference was reduced among patients with painful diabetic polyneuropathy [9]. On the contrary, exercise has been reported to improve the severity of symptoms in patients with diabetic nephropathy and neuropathy, but there have been no studies on its effect in patients with retinopathy [10,11]. The reason for this finding is that neovascularization is a problem in retinopathy, and the vulnerability of these vessels is thought to increase the risk of hemorrhage with an exercise-induced increase in blood pressure [5]. A point to consider during exercise in retinopathy is the association of proliferative diabetic retinopathy with neovascular glaucoma (NVG) [12,13]. NVG is an eye disease in which ischemia of the retina causes the formation of new blood vessels in the eye, resulting in an increased IOP. Therefore, the clarification of exercise-induced IOP fluctuation may be important in glaucoma and other eye diseases.

On the other hand, few studies have been conducted on IOP changes during exercise. In a previous study that evaluated IOP before and after 60–80 watts of moderate-intensity aerobic exercise, both normal subjects and patients with glaucoma showed a significant reduction in IOP after exercise [14]. In another study on resistance exercise, Vieira et al. investigated changes in IOP in healthy subjects during weightlifting. The exercise was performed at 80% of the one repetition maximum for four repetitions, and the IOP was measured. The results showed an increase of 2.2 ± 3.0 mmHg [15]. However, there are no detailed reports on the effects of different intensities of aerobic exercise on changes in IOP. Therefore, this study aimed to clarify the effects of low-, moderate-, and high-intensity exercise on IOP in healthy subjects.

## 2. Materials and Methods

### 2.1. Participants

This study included 18 healthy male subjects who agreed to participate in the study and were diagnosed as healthy by a rehabilitation doctor before the study (age, 24.6 ± 2.7 years; height, 172.0 ± 5.0 cm; bodyweight, 62.9 ± 5.9 kg; and BMI, 21.2 ± 1.1 kg/m^2^). The exclusion criteria were as follows: past and present smoking habits, musculoskeletal diseases that made exercise difficult, cardiovascular diseases, eye diseases under treatment, and a resting IOP < 10 mmHg or >21 mmHg. Written consent from each participant was obtained before the study. The study was approved by the Epidemiological Ethics Review of Hiroshima University (E-529).

### 2.2. Measurement Protocol

On the first day, the submaximal cardiopulmonary exercise test was performed. An expiratory gas analyzer (aero monitor: AE-310s, Minato Medical Science Co., Ltd., Osaka, Japan) and a bicycle ergometer (strength ergometer: BK-ERG-003, Mitsubishi Engineering Co. Ltd., Tokyo, Japan) were used to assess the oxygen uptake at each stage and calculate the maximal oxygen uptake by extrapolation. At intervals of at least 1 day, aerobic exercise on a bicycle ergometer was performed for 20 min at 30% ·VO_2_, 50% ·VO_2_, and 70% ·VO_2_, and IOP was assessed at rest and during exercise. Each exercise session was performed at intervals of at least 1 day. All measurements were performed from 19:00, as IOP has diurnal fluctuations.

### 2.3. Maximal Cardiopulmonary Exercise Test

The submaximal exercise stress test, performed using an expiratory gas analyzer and a bicycle ergometer, was used to measure the maximal oxygen uptake. After 3 min of rest, the subjects underwent a four-step exercise load test, with a single step of 50 W for 3 min and a recovery step of 3 min. From rest to the end of the exercise, oxygen uptake was measured by an expiratory gas analysis, and an electrocardiogram (Bedside monitor: BSM-2301, Nihon Kohden Co., Ltd., Tokyo, Japan) and automatic blood pressure monitor (EBP-300, Minato Medical Science Co., Ltd., Osaka, Japan) were used to monitor the circulatory dynamics.

### 2.4. Constant Aerobics Exercise Protocol

**Figure 1:**
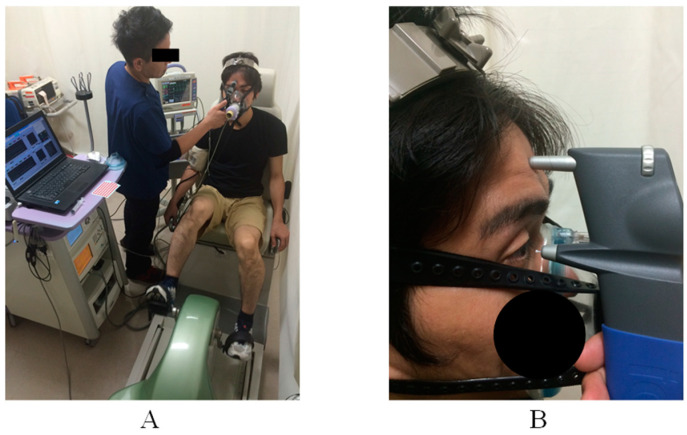
(**A**) Measurement of intraocular pressure (IOP) during exercise. (**B**) Probe position at rest and during exercise measuring IOP.

Aerobic exercise trials of three different intensities were performed at least 1 day apart from the submaximal constant exercise test. Constant exercise comprised 3 min of rest, 20 min of exercise, and 3 min of recovery. The exercise intensities were 30%, 50%, and 70% ·VO_2_ of the maximal oxygen uptake that was calculated from the submaximal exercise test, and these were performed randomly. Exercise intensity was monitored using an expiratory gas analyzer. Blood pressure and IOP measurements were taken at rest, every 5 min of exercise, and during recovery (Figure 1A).

### 2.5. Oxygen Consumption

The maximal oxygen uptake was calculated from the oxygen uptake (x) at 3 min and the heart rate (y) at each stage of the multistage exercise test. A linear regression line was constructed, and the oxygen uptake when the maximum heart rate (220—age) was substituted into the equation was used as the maximal oxygen uptake. The oxygen uptake was calculated for each minute of quantitative exercise from rest to the end of the exercise.

### 2.6. Intraocular Pressure

IOP was measured in the right eye using a handheld tonometer (iCare: TA01i, M.E. Technica, Tokyo, Japan). The tonometer was fixed on the subject’s forehead and the probe was placed perpendicular to the apex of the cornea. Two measurements were taken, and the average of the two measurements was used. Measurements were taken at rest, every 5 min of exercise, and during recovery (Figure 1B).

### 2.7. Statistical Analysis

SPSS^®^ version 25 (SPSS, Chicago, IL, USA) was used for all statistical analyses. Dunnett’s test was used to compare the dynamic changes in IOP, mean blood pressure (MBP), and ·VO_2_ during rest at each stage of exercise. Pearson’s correlation coefficient was used to correlate IOP with MBP and ·VO_2_. The statistical significance was set at *P* < 0.05.

## 3. Results

All subjects were able to perform the multistage exercise test with a ·VO_2max_ of 53.4 ± 8.0 mL/kg/min. The oxygen uptake during exercise was significantly higher at all intensities from 5 to 20 min of exercise than that at rest. MBP was significantly higher at 70% intensity from 5 to 20 min of exercise than that at rest. IOP was significantly lower at 70% intensity from 5 to 20 min of exercise than that at rest (Table 1).

**Figure 2:**
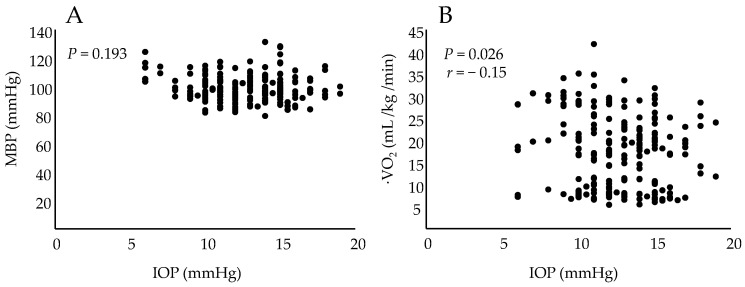
(**A**) Relationship between intraocular pressure and mean arterial pressure (MBP). (**B**) Relationship between intraocular pressure and oxygen consumption during exercise (VO_2_).

There was no correlation between IOP and MBP (*P* = 0.193), but there was a negative correlation between IOP and ·VO_2_ (*r* = −0.15, *P* = 0.026) (Figure 2).

## 4. Discussion

In the present study, we investigated the effects of low-, moderate-, and high-intensity exercise on IOP in healthy subjects. The results showed that there was no change in IOP during low- and moderate-intensity exercise compared with that at rest. However, during high-intensity exercise (70% ·VO_2_), IOP was significantly lower than that at rest until 20 min after the start of exercise, and IOP was correlated with ·VO_2_, an index of exercise intensity. Shapiro et al. investigated IOP changes before and after exercise using a bicycle ergometer at different intensities using absolute exercise intensity in watts and reported that load amount did not correlate with changes in IOP [16]. When prescribing exercise at absolute exercise intensity, the load is constant, but oxygen uptake and the circulatory response during exercise for each individual is different. Therefore, it is likely that the effective limit, which is important when prescribing exercise, will be lowered, or that the safe limit will be exceeded. Therefore, it is necessary to investigate the relationship between IOP and relative exercise intensity more accurately. In the present study, exercise intensity was observed by adjusting the load so that oxygen uptake was constant during exercise to demonstrate the relationship more accurately between exercise intensity and IOP. The results of this study may also apply to general clinical practice, as exercise prescriptions are generally based on relative exercise intensity using the Karvonen or other methods [17].

IOP is generated by the aqueous humor and describes the pressure that is experienced by the entire closed system. Aqueous humor is produced by the ciliary epithelium and drains through two independent outflow pathways: the trabecular meshwork and the uveal tract. Recently, it was reported that uveal lymphatics are also involved in IOP regulation [18]. The decrease in aqueous humor, which is related to IOP, is due to the inhibition of NO synthesis [19], and β-blockers, which have long been used to reduce IOP in glaucoma [20]. As observed in this study, exercise is not a factor in lowering IOP due to NO production and increased sympathetic nerve activity, suggesting that other mechanisms may be involved in aerobic exercise. Hariss et al. evaluated the relationship among IOP, PCO_2_, and plasma osmolality after 7 min of exercise at 30 W and 90 W on a bicycle ergometer, followed by gradual increases in exercise intensity. The results showed that PCO_2_ and plasma osmolality were not related to the decrease in IOP; however, there was a negative correlation between IOP and lactate levels [21]. In the present study, IOP decreased with high-intensity exercise. There was no relationship between IOP and MBP but a negative correlation between ·VO_2_ and IOP, and this might have been due to the rapid increase in lactate levels in high-intensity exercise.

In previous studies of transient exercise and training in healthy volunteers, optical coherence tomography angiography revealed a reduction in the blood flow in the optic papilla and macula [22,23]. The effects of exercise intensity on optic nerve and macular fundus perfusion in patients with retinopathy, glaucoma, and other ocular diseases are not clear. Therefore, fundus blood flow, including IOP, should be investigated to determine the safe level of exercise for patients with various ocular diseases.

The first limitation of the present study is that the data were derived exclusively from healthy men. However, IOP reportedly varies according to sex and age [24]. Therefore, future studies should take these differences into account by including a more diverse cohort of individuals. A second limitation is that we did not measure the lactate level, which is an IOP-lowering factor. Therefore, future studies should include this parameter.

In healthy participants, IOP did not change during low- and moderate-intensity exercise, but significantly decreased during high-intensity exercise compared with that at rest. The results suggest that different mechanisms are involved in the lowering of IOP at rest and during exercise.

## Figures and Tables

**Table 1:** Differences in intraocular pressure, blood pressure, and oxygen uptake at each exercise intensity.

	30%-intensity aerobic exercise					
	Rest	5 min	10 min	15 min	20 min	Recovery
IOP (mmHg)	13.7 ± 2.2	13.0 ± 2.2	12.9 ± 2.2	12.4 ± 2.4	12.1 ± 2.3	12.5 ± 2.9
MBP (mmHg)	93.2 ± 9.5	97.6 ± 6.3	95.2 ± 8.7	95.5 ± 7.1	95.0 ± 8.5	93.6 ± 7.1
·VO_2_ (mL/kg/min)	5.5 ± 1.5	8.7 ± 2.7 *	8.4 ± 2.7 *	8.6 ± 2.9 *	8.6 ± 2.7 *	5.7 ± 1.5 *
	50%-intensity aerobic exercise					
	Rest	5 min	10 min	15 min	20 min	Recovery
IOP (mmHg)	14.3 ± 2.7	13.9 ± 2.5	13.1 ± 2.7	13.4 ± 2.9	13.1 ± 3.3	13.0 ± 2.9
MBP (mmHg)	93.7 ± 11.2	97.5 ± 7.1	95.7 ± 7.9	97.3 ± 8.1	96.8 ± 9.9	93.6 ± 8.3
·VO_2_ (mL/kg/min)	5.7 ± 1.6	17.7 ± 4.3 *	17.6 ± 4.4 *	16.5 ± 3.8 *	15.8 ± 4.3 *	7.8 ± 2.6
	70%-intensity aerobic exercise					
	Rest	5 min	10 min	15 min	20 min	Recovery
IOP (mmHg)	14.2 ± 2.6	12.4 ± 2.8 *	11.5 ± 2.6 *	11.5 ± 2.5 *	11.6 ± 2.8 *	13.1 ± 2.3
MAP (mmHg)	94.3 ± 10.4	110.0 ± 12.4 *	103.3 ± 9.9 *	102.2 ± 7.5 *	100.3 ± 7.1 *	94.1 ± 11.2
·VO_2_ (mL/kg/min)	5.8 ± 1.7	28.7 ± 5.2 *	25.7 ± 4.7 *	24.6 ± 3.9 *	24.6 ± 4.3 *	7.0 ± 2.0

Mean ± standard deviation. * Statistically significant (*P* < 0.05). IOP, intraocular pressure; MBP, mean blood pressure; ·VO_2_, oxygen consumption during exercise.

## Data Availability

Data are not available to the public.
